# Robustness of Tomato Quality Evaluation Using a Portable Vis-SWNIRS for Dry Matter and Colour

**DOI:** 10.1155/2017/2863454

**Published:** 2017-12-03

**Authors:** U. K. Acharya, P. P. Subedi, K. B. Walsh

**Affiliations:** Central Queensland University, Building 361, Rockhampton, QLD 4701, Australia

## Abstract

The utility of a handheld visible-short wave near infrared spectrophotometer utilising an interactance optical geometry was assessed in context of the noninvasive determination of intact tomato dry matter content, as an index of final ripe soluble solids content, and colouration, as an index of maturation to guide a decision to harvest. Partial least squares regression model robustness was demonstrated through the use of populations of different harvest dates or growing conditions for calibration and prediction. Dry matter predictions of independent populations of fruit achieved *R*^2^ ranging from 0.86 to 0.92 and bias from −0.14 to 0.03%. For a CIE *a*^⁎^ colour model, prediction *R*^2^ ranged from 0.85 to 0.96 and bias from −1.18 to −0.08. Updating the calibration model with new samples to extend range in the attribute of interest and in sample matrix is key to better prediction performance. The handheld spectrometry system is recommended for practical implementation in tomato cultivation.

## 1. Introduction

Tomato fruit quality is assessed in terms of dry matter content (DM, also known as total solids content), soluble solids content (SSC), and external colour, and less commonly in terms of firmness and titratable acidity [1, 2]. There is also interest in the level of lycopene, given suggested health benefits [3].

Starch is stored in immature fruit, with conversion to reducing sugars (e.g., glucose and fructose) around 40 days after anthesis [4]. In fruit near maturity, the DM of tomato pulp consists of soluble sugars (50% w/w), insoluble solids (25%), organic acids (13%), minerals (8%), and others (4%) [5]. In the tomato processing industry, grower payments may be linked to DM content of pulp, as processing to products such as ketchup involves concentration of soluble solids content. Maximisation of fruit solids content is a matter of maximising carbohydrate import to the fruit, through use of appropriate genetic material (varieties), growing conditions, and agronomic practices [2]. For example, the assessment of fruit for DM and soluble solids content is practiced in varietal screening programs [6]. It is also well established that fruit of plants exposed to moderate salinity will accumulate a higher level of solids [7]. Gauging the appropriate time of harvest is a key agronomic decision, balancing the need to leave the fruit on vine to maximise solids content, and the need to harvest fruit before ripening proceeds to the point where loss of firmness means that the fruit will be damaged during harvest, transport, and processing.

Near infrared spectroscopy (NIRS) is a potential tool for nondestructive measurement of several fruit attributes. NIRS assessment of intact fruit (vegetable) DM content was first reported for onion [8]. Numerous reports have subsequently been produced for use of this technology with a range of fruit commodities; however many studies are compromised either in terms of matching the optically sampled volume to the volume of tissue sampled for reference analysis, or in terms of overfitting of a multivariate model, without testing on a truly independent population. Nonetheless, the technology is in commercial use, both for in-line sorting of fruit operating on conveyor systems moving at up to 1 m/sec and for assessment of fruit on tree, based on use of handheld near infrared spectrometers, for example, in noninvasive assessment of DM of mango fruit on tree [9]. Such noninvasive data is of use in decision support systems around assessment of fruit maturation or ripening (e.g., Jordan and Loeffen [10]).

The first consideration of the use of NIRS to assess intact tomato soluble solids content (SSC) was made by Slaughter et al. [11] using a laboratory bench top system (NIR System 6500) using fibre optics in an interactance geometry. Spectra were acquired at four equatorial positions and at the acropetal end of fruit. Partial least squares regression (PLSR) models based on spectra from the equatorial region were better than that from acropetal end in terms of correlation coefficient and root mean square of errors in calibration (RMSEC). Based on a 800–1000 nm spectral window and use of 12 PLS factors (*f*), *R*^2^ of 0.85 and RMSEC on SSC of 0.27% were achieved, with validation statistics of *R*^2^ = 0.79, root mean square of error of prediction (RMSEP) of 0.33%, and bias of 0.05%. However, the validation set was not independent of the calibration population (of 400 spectra initially taken, 300 were used as training and 100 as prediction set).

Subsequently, a number of researchers have considered use of NIR/IR spectroscopy for assessment of quality parameters of tomato juice, puree, or intact fruit [12–14]. These studies have involved a range of equipment, varying in wavelength range, wavelength dispersion mechanism, and optical geometry employed (reflectance, diffusive reflectance, transmittance, or interactance), in data preprocessing (first derivative, second derivative, and multiplicative scatter correction, MSC), and in the calibration model development method (principal component regression, PCR; multiple linear regression, MLR; PLSR1; PLSR2; and modified partial linear square, MPLSR). For example, for a set of transmission spectra of intact fruit acquired with a MMS1 NIR spectrometer, Khuriyati and Matsuoka [15] reported that PLSR models of SSC were better when based on second derivative spectra (*R*^2^*c* = 0.92, RMSEC = 0.36%) than smoothed log (1/*T*) spectra. However, this study lacks documentation of regression coefficients (the smoothness of which allows a judgement of likely model robustness), and again the validation and calibration sets were selected from the one population (i.e., the validation set is not independent of the calibration set).

In general there has been a shift from use of reflectance to interactance or partial transmittance geometries. Indeed, Clement et al. [16] severely criticised use of reflectance spectra for SSC estimation of intact fruit, due to poor validation performance.

A number of workers have used a spectral range of 900 nm and above for PLSR calibration models of tomato juice and puree SSC [13, 14, 17], but Shao and He [18] attempted use for intact fruit. As the longer wavelengths have high absorbance by water, the effective depth of optical assessment is low (1-2 mm) and thus effectively only the skin will be assessed. It is expected that this model will lack robustness; that is, it will perform relatively poorly in prediction of independent populations of intact fruit that vary in skin properties.

The Herschel wavelength region (720–1100 nm, also known as the short wave NIR or SWNIR) is typically employed due to greater effective penetration through biological tissue and thus optical sampling volume than for longer wavelengths. The miniature Zeiss MMS1 Si photodiode array spectrometer operates over the wavelength range 300–1100 nm and has seen used in several studies involving tomato. For example, in common with Khuriyati and Matsuoka [15] and Walsh et al. [19], Khuriyati et al. [12] used this spectrometer in development of a dry matter PLS model on tomato DM, based on interactance spectra. A coefficient of determination (*R*^2^) of 0.88 was achieved, but again the model was not tested on independent fruit populations. Most recently, Radzevicius et al. [20] reported the use of the luggable NIR Case NCS001A (Sacmi Imola S.C., Italy) for estimation of tomato DM, SSC, and firmness, but calibration statistics were not presented.

Other limitations of past studies include a low standard deviation (SD) in the attribute of interest in the training or validation set, as a low range in the population will necessarily be associated with an apparently low RMSEC and a low *R*^2^. For example, poor SSC model performance for a data set based on interactance SWNIR spectra of intact tomato fruit was due to a population SD of only 0.3% [19].

The measurement of tomato colour using portable SWNIR instrumentation (FQA-NIRGUN, interactance optics, 623–1052 nm) was reported by Kusumiyati et al. [21] for tomato both on and off vine, with better results achieved using a PLSR than a PCR model. For the PLSR model of CIE *a*^*∗*^, calibration (*R*^2^ = 0.92; RMSEC = 3.31; *f* = 4) and prediction statistics of *R*^2^ = 0.92, SEP = 3.19, and RPD (ratio of bias corrected SEP to standard deviation) of 4.76 were reported. Clement et al. [16] assessed tomato colour (Hunter a) based on reflectance spectra (400–1500 nm) of intact tomato acquired using a Varian Cary 500 UV-Vis-NIR spectrophotometer (Varian Inc., Palo Alto, CA). PLSR model statistics for Hunter a were *R*^2^ = 0.981, RMSECV = 1.126, and standard deviation ratio (SDR, a ratio of RMSECV to SD) = 7.32. The spectral window of 400–1000 nm produced a better result than the 900–1500 nm window, as expected for this attribute. More recently, Camps [22] reported the use of a handheld diffuse reflectance NIR spectrometer using the 900–1700 nm region (Phazir; Analyticon Instruments, Germany) to assess CIE *L*^*∗*^ and *a*^*∗*^ values of tomato, reporting a calibration *R*^2^_*c*_ of 0.903 and 0.846 and RMSECV of 2.26 and 3.02, respectively. It would have been interesting to see comparative results based on the visible-SWNIR region, and prediction of independent test sets.

Thus previous reports indicate the potential of SWNIRS in a reflectance geometry for assessment of intact tomato colour, and interactance or transmission geometries for DM and SSC assessment. A number of studies refer to the potential for field portable instruments, allowing application to the agronomy of the crop. However, a consistent criticism of all previous studies is the use of one population divided into a calibration and a “validation” set, with validation samples often deliberately chosen to be representative of the calibration set (i.e., same mean and SD). Such validation sets are not independent of the calibration set and the validation statistics can be expected to be optimistic in terms of the performance of the model in prediction of future predictions, as must occur in practical use. The current study is undertaken to evaluate the use of portable SWNIRS for field assessment of colour and DM of intact tomato, involving a rigorous verification study across truly independent validation sets.

## 2. Materials and Methods

### 2.1. Plant Culture

One hundred four-week old tomato (*Lycopersicum esculentum* L., var. Roma) seedlings were transplanted to a hydroponic system consisting of 10 L plastic containers (single plant per container), connected to an irrigation system to supply nutrient solution. The base nutrient solution consisted of a solution of electrical conductivity of 1.36 mS cm^−1^ and pH 6.0. Temperature of the solution varied between 25 and 31°C throughout the trial period. To induce a range in fruit dry matter and colour, several treatments were imposed.

At fruit bearing stage, plants were assigned into five treatment groups, each group with four replicates (i.e., 20 plants per treatment) in a randomized complete block design. The five treatments were (i) plants on normal hydroponic solution (base), (ii) base with added KNO_3_ (198 mg L^−1^; final solution conductivity of 1.6 mS cm^−1^), (iii) base with added NaCl (2 g L^−1^ final solution conductivity of 5.5 mS cm^−1^), (iv) base with leaf removal (to one leaf/truss), and (v) base with fruit removal (to four fruit/truss). For the KNO_3_ and NaCl treatments, salt was added to the base solution once the plant started producing flowers, and the leaf and fruit removal treatments were started when fruitlets were visible. Four weeks after flowering, the strength of the normal hydroponic solution was increased to 1.63 mS cm^−1^ while NaCl and KNO_3_ treated solution were increased to 6.1 and 1.93 mS cm^−1^, respectively.

### 2.2. Fruit Measurement

Visible-SWNIR spectra of intact fruit were acquired with a “Nirvana” SWNIR spectrometer (no longer produced, but equivalent to the F750, Felix Instruments, WA, USA). This unit employs an interactance geometry, a halogen lamp, and a Zeiss MMS1 Si photodiode array with approximately 3.3 nm pixel spacing and 10 nm wavelength resolution. The interactance probe involves a receiving optical probe placed in front of a collimated light source, such that the probe casts a shadow onto the sample and the probe views the cast shadow on the sample [23]. Thus detected light has passed through part of the fruit.

Five sets of tomato fruit (termed populations 1 to 5) were harvested at intervals of 10 days from the seventh week after planting from all five growing condition treatments. On each harvest date, two plants were chosen at random from each treatment and all fruit were harvested from first to fourth trusses (resulting in between 50 and 215 fruit in each harvest, Table 1). These fruit were numbered in the field, brought into the laboratory (at 22°C), and marked at a location on the fruit equatorial region. The SWNIR spectrometer was used to collect interactance spectra, in duplicate, at each marked spot. A spot colour reading in CIE1976 *L*^*∗*^*a*^*∗*^*b*^*∗*^ space was made at the same position with a Minolta colorimeter (CR-400, D65 illuminant, 2° angle of observer). Following these measurements, a one centimetre sided cube of fruit flesh from the point of scanning was collected, diced, and placed in a forced air oven at 65°C for 48 hour for dry matter estimation.

To emulate field use of the handheld spectrometer in monitoring of fruit maturation, five plants were randomly selected from each of the five treatments (total *n* = 25) and four fruit per plant (at least one fruit from each of second to fourth truss) were tagged (total *n* = 100 fruit). The tagged fruits were scanned with the handheld spectrometer at a position on the fruit equator. Scanning was repeated each week of the same location on each fruit, from sixth week after planting until most of the fruit were fully red ripe (11 weeks after planting).

### 2.3. Data Preparation and Analysis

The absorbance spectral data acquired from handheld spectrometer were transformed to (3 nm) interpolated second derivative (Savitzky-Golay second-order, 7 point window) spectra (referred to as “second derivative” spectra). Partial least squares regression models were developed using the multivariate data analysis software (The Unscrambler V 10.2, Camo Inc., Norway). Models were developed using the NIPALS (nonlinear iterative partial least squares) algorithm and were cross validated with random subsets of four samples.

DM and CIE *a*^*∗*^ models were developed using population 1 and “updated” by sequential expansion of the calibration set with populations 2 to 5. These PLSR models were tested on separate fruit populations (i.e., model based on population 1 used in prediction of population 2, and model based on populations 1 and 2 used in prediction of population 3, etc., with the model based on populations 1 to 5 used in prediction of the populations of fruit from the five growing conditions).

## 3. Results and Discussion

### 3.1. Spectral Features

**Figure 1:**
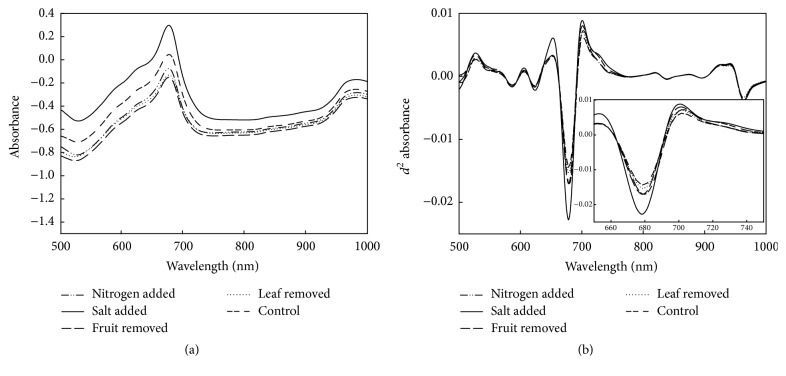
(a) Absorbance spectra and (b) second derivative of absorbance spectra of fruit from five growing conditions (average of 10 fruit) at 7 weeks after planting. Inset shows magnification of 660–740 nm range.

Fruit absorbance spectra were characterised by a peak at 675 nm ascribed to chlorophyll, a peak ascribed to lycopene at 575 nm, and peaks ascribed to O-H vibrations of water at 840 and 960 nm (Figures 1(a) and 1(b)). As fruit matured, absorbance spectra were characterised by a decrease in the positive peak at 675 nm related to chlorophyll and an increase in a peak related to lycopene at 575 nm. Similar trends in peak changes related to pigment and water features were also reported by Clement et al. [16].

### 3.2. Calibration Model

**Figure 2:**
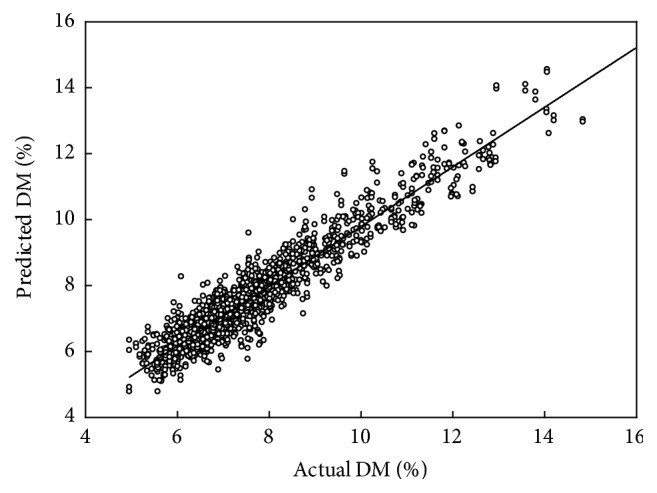
Scatter plot of PLSR model (cross validation) predicted and actual values for DM (*R*^2^_cv_ = 0.902, RMSECV = 0.512, and *n* = 1585) for the combined population set.

Dry matter was better modelled using second derivative than absorbance spectra (data not shown), with a resulting cross validation coefficient of determination (*R*^2^_cv_) ranging from 0.90 to 0.93 and a RMSECV < 0.5% and a ratio of RMSECV to population SD > 3.0 (Table 1, Figure 2). The intact tomato DM models were superior to that reported by Walsh et al. [19] due to the poor standard deviation (SD) of the population employed in that study (0.30, compared to more than 1.5% in the current study). The results were also superior to that reported by Khuriyati and Matsuoka [15] although that study employed a population with SD = 1.84% for DM and used short wave NIR in an interactance geometry. As expected, the result was inferior to that for tomato puree DM model from mid-infrared reflectance spectra as reported by Ścibisz et al. [14], given the influence of fruit skin on intact fruit spectra. The PLSR model *b*-coefficient weightings were consistent across models (based on different treatments/populations), with weighting around 830, 880, and 910 nm (Figure 4), similar to that reported by Walsh et al. [19], and consistent with involvement of the second overtone of O-H, fourth overtone C-H, and the third overtone feature of C-H, respectively [24, 25]. The presence of relatively “smooth” *b*-coefficients, consistent across models, bodes well for the robustness of the model in prediction of independent sets.

**Figure 3:**
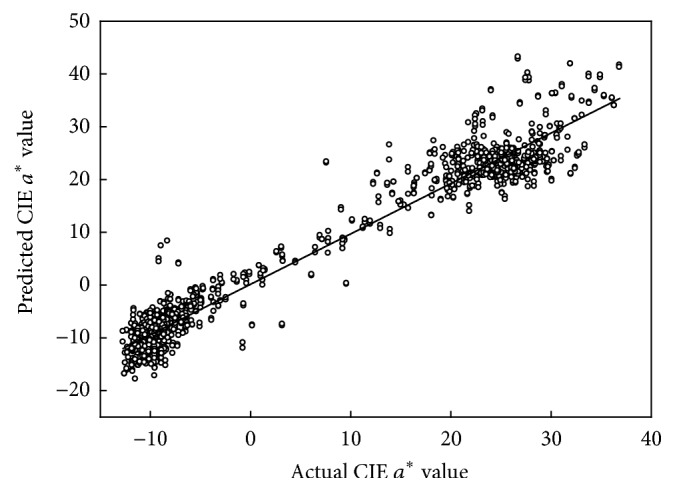
Scatter plot of PLSR model (cross validation) predicted and actual values for colour CIE *a*^*∗*^ (*R*^2^_cv_ = 0.956, RMSECV = 3.35, and *n* = 1567) for the combined population set.

**Figure 4:**
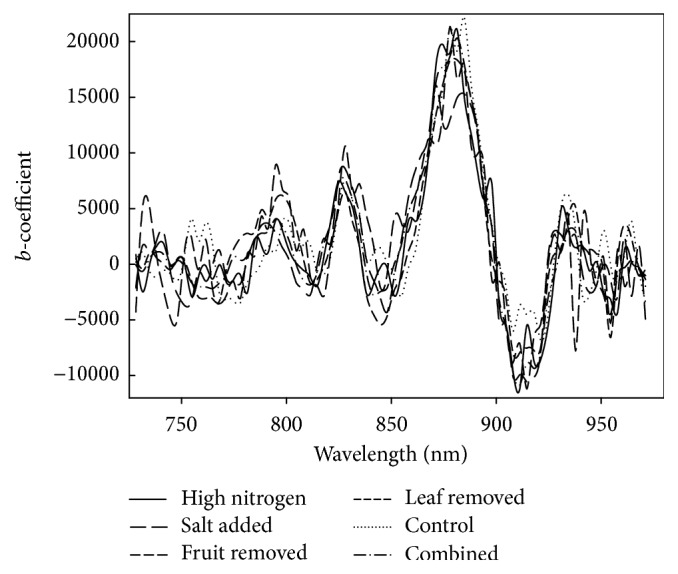
PLSR model *b*-coefficients for DM for models based on individual and combined treatment populations.

**Figure 5:**
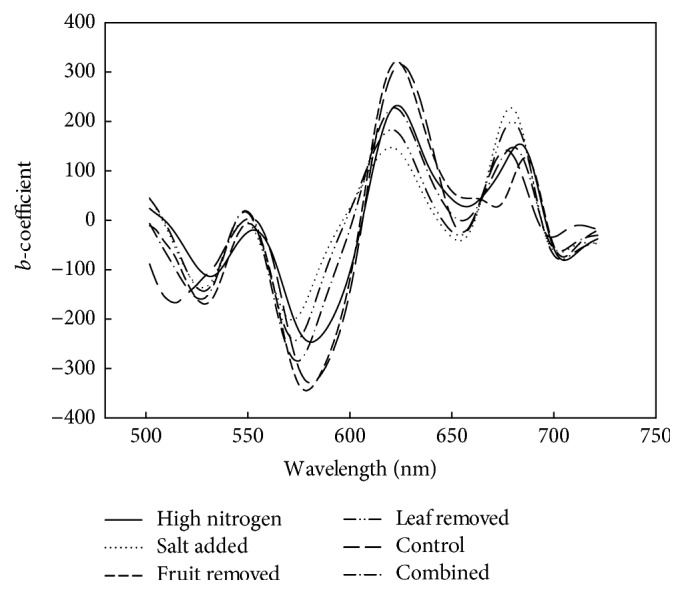
PLSR model *b*-coefficients for CIE *a*^*∗*^, for models based on individual and combined treatment populations.

The colour (CIE *a*^*∗*^) model based on second derivative spectra was poor for a data set involving population 1 only, due to the low attribute range in immature, green fruit (Table 1). Subsequent populations modelled well for CIE *a*^*∗*^, with *R*^2^ > 0.9, RMSECV~3.5, and SDR > 4 (Figure 3), although for one population (Pop 2) an excessive number of factors was adopted. The colour PLSR model possessed a negative weighting at 500–600 nm and positive weighting between 600 and 720 nm (Figure 5). As for DM models, the CIE *a*^*∗*^ model *b*-coefficients were consistent across models based on different populations.

### 3.3. Robustness of Calibration Model

Previous research reports have separated a subset of a given population for use in validation of a prediction model developed on the remainder of the population. In commercial practice, an existing model must be used in prediction of an incoming population, not included in the calibration set. Samples from the new population may be used to update the model, in preparation for prediction of new sets. To emulate this practice, a model was developed and used in prediction of an independent set, and then this combined data set was used to create a new model which was used in prediction of another set of fruit.

A single population (Pop 1) DM model was relatively robust, with *R*^2^ = 0.84, RMSEP = 0.86, and bias = −0.26% in prediction of fruit from a subsequent harvest week (Table 2); however, a DM model based on combined populations (Figure 2) was more effective. For example, population 5 was predicted well by a model based on populations 1 to 4, with *R*^2^ = 0.92, RMSEP = 0.5, and bias = 0.03. A model used to predict DM of fruit from a given agronomic treatment achieved a lower *R*^2^ than for the time series predictions, due to the lower SD of these validation sets. In general, prediction accuracy (RMSEP) of 0.5% was achieved, with a bias less than 0.2% (Table 2).

Tomato colour is determined by the content of pigments, that is, chlorophyll and lycopene, in the fruit mesocarp. Given the thin skin of a tomato, the colour measurements made using the diffuse reflectance optics of the Minolta colorimeter will gather information from a depth of several millimetres into the fruit, while the interactance optics of the SWNIR spectrometer will collect information to a greater depth (ca. 10–20 mm). However, given the good performance of the SWNIR instrument based models created using reference data from the Minolta unit, the tissue volumes sampled by the two instruments must have similar pigmentation. CIE *a*^*∗*^ models based on a single or few populations were poor in prediction performance as these populations include a narrow colour range in the calibration set (Table 2). With addition of fruit of all colour stages in the calibration set, the prediction results were acceptable. For example, prediction statistics for a model based on populations 1 to 4 used in prediction of population 5 and of fruit subject to various agronomic treatments included *R*^2^ values from 0.82 to 0.95 and a bias of −1.18 to 0.67.

**Figure 6:**
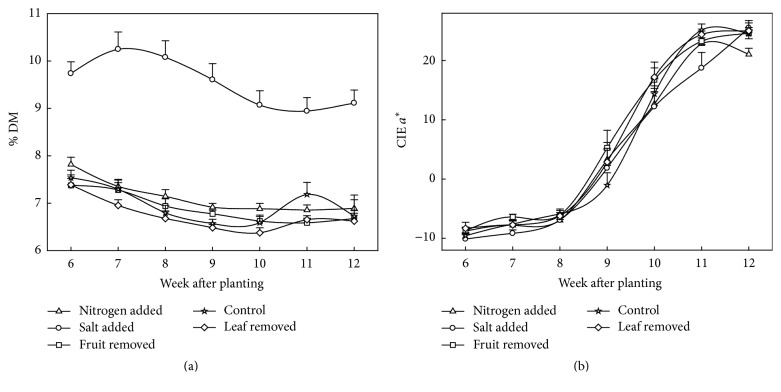
Change in PLSR model predicted (a) DM (%) and (b) colour (CIE *a*^*∗*^) over a growing period (*n* = 20, error bar represents standard error of mean).

As an example of practical implementation, PLSR models on DM and CIE *a*^*∗*^ (based on fruit grown under a range of growing conditions) were used in prediction of these attributes given spectra collected nondestructively each week from fruit of the various agronomic treatments (Figure 6). This emulates how the handheld spectrometry system could be used on farm to monitor fruit maturation.

## 4. Conclusion

A handheld interactance geometry visible-SWNIR spectrophotometer suitable for field practice was used to judge maturity of tomato fruit based on pigmentation as indexed by CIE *a*^*∗*^ and eating quality, as indexed by DM, both on and off vine. PLSR models based on visible wavelengths were robust in prediction of new data sets, with low bias, *R*^2^ > 0.75 and 0.82, and RMSEP < 0.84 and 6.88 on the attributes of DM (%) and CIE *a*^*∗*^, respectively. The range of DM in fruit from plants under a given growing condition was relatively limited, so use of a range of growing conditions to achieve a wide range of fruit DM is recommended for DM model development. Fruit of different maturities from a given growing condition is adequate for providing a range of colour. “Updating” a calibration model by addition of current samples to the training population to extend the range of the attribute of interest and the range of chemical and physical matrices to be found associated with tomato fruit is recommended to achieve robust practical use. This technology should have value in tomato breeding programs as a selection tool, to hydroponic producers wishing to increase eating quality by manipulation of solution conductivity, and as a quantitative tool in estimating time to harvest. Time courses of attribute development (Figure 6) can be used to inform crop management.

## Figures and Tables

**Table 1:** Population and calibration model statistics of hydroponic tomato fruit DM (729–975 nm) and colour (CIE *a*^*∗*^) (500–720 nm), based on the interactance spectra (#*f* refers to the number of PLS factors).

Population	DM population statistics			DM PLSR model statistics				CIE *a*^*∗*^ population statistics		CIE *a*^*∗*^ PLSR model statistics			
	Sample #	Mean	SD	#*f*	*R* ^2^_cv_	RMSECV	SDR	Mean	SD	#*f*	*R* ^2^_cv_	RMSECV	SDR
Pop 1	360	7.67	1.52	9	0.91	0.43	3.55	−9.92	1.12	2	0.69	0.6	1.86
Pop 2	420	7.93	1.99	9	0.94	0.49	4.04	−4.54	12.65	7	0.98	1.62	7.83
Pop 3	430	7.7	1.67	8	0.91	0.47	3.54	6.59	16.18	2	0.88	5.64	2.87
Pop 4	396	7.65	1.48	9	0.91	0.45	3.37	17.99	12.64	1	0.96	3.46	3.65
Pop 5	100	8.26	1.87	8	0.91	0.54	3.47	16.17	15.67	2	0.95	3.55	4.42
Pop 1-2	780	7.81	1.79	10	0.92	0.48	3.72	−7.02	9.69	2	0.96	1.79	5.41
Pop 1–3	1210	7.77	1.75	10	0.9	0.52	3.37	−2.18	13.99	2	0.93	3.54	3.95
Pop 1–4	1606	7.74	1.68	10	0.9	0.51	3.28	2.79	16.2	2	0.95	3.65	4.44
Pop 1–5	1706	7.77	1.7	10	0.93	0.52	3.28	3.59	16.48	2	0.95	3.49	4.72
N added	484	7.13	1	11	0.84	0.38	2.6	2.82	15.56	2	0.93	4.08	3.82
Salt added	280	10.53	1.68	10	0.91	0.49	3.43	7.29	18.37	2	0.96	3.62	5.07
Fruit removed	300	7.59	0.95	11	0.84	0.36	2.67	2.08	15.63	2	0.97	2.49	6.26
Leaf removed	748	7.29	1.48	9	0.88	0.51	2.88	9.94	16.33	2	0.93	4.21	3.88
Control	362	7.5	1.13	11	0.87	0.38	2.95	2.36	16.13	2	0.94	3.85	4.19

**Table 2:** Prediction statistics of hydroponic tomato fruit DM (729–975 nm) and colour (CIE *a*^*∗*^) (500–720 nm), based on interactance spectra.

Populations		DM				CIE *a*^*∗*^			
Calibration set	Prediction set	Bias	*R* ^2^_*p*_	RMSEP	SDR	Bias	*R* ^2^_*p*_	RMSEP	SDR
Pop 1	Pop 2	−0.26	0.84	0.86	2.32	−5.2	0.17	13.07	0.97
Pop 1-2	Pop 3	0.07	0.86	0.62	2.70	−2.7	0.73	8.87	1.82
Pop 1–3	Pop 4	−0.14	0.87	0.56	2.64	−0.48	0.85	5.00	2.5
Pop 1–4	Pop 5	0.03	0.92	0.53	3.56	−1.18	0.95	3.80	4.09
Pop 1–5	N added	0.19	0.76	0.55	1.83	0.67	0.82	6.88	2.26
Pop 1–5	Salt added	−0.19	0.76	0.84	2.00	−0.29	0.95	4.13	4.45
Pop 1–5	Fruit removed	0.03	0.77	0.46	2.07	−0.08	0.95	3.02	5.18
Pop 1–5	Leaf removed	−0.03	0.88	0.51	2.88	0.11	0.86	6.01	2.72
Pop 1–5	Control	−0.18	0.83	0.49	2.29	−0.54	0.78	7.64	2.11

## Additional Points

*Highlights.* (i) Application of a handheld visible-NIR spectrometer using an interactance geometry to judge maturity and quality of tomato fruit based on colour (CIE *a*^*∗*^) and DM, both on and off vine, is demonstrated. (ii) Dry matter predictions achieved *R*^2^ ranging from 0.86 to 0.92 and bias from −0.14 to 0.03%. For CIE *a*^*∗*^, prediction *R*^2^ ranged from 0.85 to 0.96 and bias from −1.18 to −0.08.
